# Predicted Enhanced Human Propensity of Current Avian-Like H1N1 Swine Influenza Virus from China

**DOI:** 10.1371/journal.pone.0165451

**Published:** 2016-11-09

**Authors:** Veljko Veljkovic, Nevena Veljkovic, Slobodan Paessler, Marco Goeijenbier, Vladimir Perovic, Sanja Glisic, Claude P. Muller

**Affiliations:** 1 Center for Multidisciplinary Research, Institute of Nuclear Sciences Vinca, University of Belgrade, Mihajla Petrovica 12-14, 11001 Belgrade, Serbia; 2 Department of Pathology, Galveston National Laboratory, University of Texas Medical Branch, 301 University Boulevard, Galveston, TX, United States of America; 3 Department of Viroscience, Erasmus Universitair Medisch Centrum Rotterdam, Rotterdam, Netherlands; 4 Department of Infection and Immunity, Luxembourg Institute of Health, 29, rue Henri Koch, L-4534 Esch-Alzette, Grand-Duchy of Luxembourg; Johns Hopkins University Bloomberg School of Public Health, UNITED STATES

## Abstract

Influenza A virus (IAV) subtypes against which little or no pre-existing immunity exists in humans represent a serious threat to global public health. Monitoring of IAV in animal hosts is essential for early and rapid detection of potential pandemic IAV strains to prevent their spread. Recently, the increased pandemic potential of the avian-like swine H1N1 IAV A/swine/Guangdong/104/2013 has been suggested. The virus is infectious in humans and the general population seems to lack neutralizing antibodies against this virus. Here we present an *in silico* analysis that shows a strong human propensity of this swine virus further confirming its pandemic potential. We suggest mutations which would further enhance its human propensity. We also propose conserved antigenic determinants which could serve as a component of a prepandemic vaccine. The bioinformatics tool, which can be used to further monitor the evolution of swine influenza viruses towards a pandemic virus, are described here and are made publically available (http://www.vin.bg.ac.rs/180/tools/iav_mon.php; http://www.biomedprotection.com/iav_mon.php).

## Introduction

Influenza A virus (IAV) infections are the major cause of serious human respiratory tract infections worldwide. The principal antigenic determinant of IAV is glycoprotein hemagglutinin (HA) on the surface of the virus that stimulates host neutralization antibody responses. There are 18 different HA subtypes which are named H1 through H18. This viral protein is synthesized as a precursor that is then glycosylated and cleaved into two smaller polypeptides: the HA1 and HA2 subunits. HA1 allows the recognition of target vertebrate cells, accomplished through the binding to these cells' sialic acid-containing receptors). HA2 mediates fusion of the host endosomal membrane with the viral membrane, allowing entry of viral ribonucleoprotein into the host cell.

IAV is well known to undergo antigenic drift escaping immunity and causing the typical seasonal flu outbreaks in humans. Antigenic shift is another evolutionary process that is responsible for new emerging IAV subtypes against which little or no pre-existing immunity exists in the human population. The introduction of a new influenza subtype may result in a rapidly spreading pandemic and a serious threat for public health. Early and rapid detection of candidate pandemic IAVs is crucial for preventing their spread, and for preparing for the production of an appropriate vaccine [1]. In the past, specific diagnostic tools, and the production of dedicated vaccines commenced only after the infection of humans and human to human transmission. The 2009 influenza pandemic caused by the novel H1N1 IAV showed the limitations of this approach. Therefore it is necessary to pro-actively monitor IAV circulating in animal hosts in order to stay ahead of a potential pandemic threat.

Pigs are important hosts for generating novel IAV. Recently, 139 IAV strains belonging to the Eurasian avian-like H1N1 swine IAV (SIV) were reported from China. This lineage circulated in pigs since 1979 and reportedly also infected humans [2]. These viruses formed two distinct antigenic subgroups represented by A/swine/Guangxi/18/2011 and A/swine/Guangdong/104/2013 SIV. It was found that 3.6% of 55 children tested, 0% of 52 adults and 13.4% of 52 elderly adults had neutralization antibodies against the A/swine/Guangxi/18/2011, and that none of them had neutralization antibodies against the A/swine/Guangdong/104/2013 virus [2]. Since the A/swine/Guangdong/104/2013 virus preferentially binds human-type receptors and is antigenically and genetically distinct from the current human H1N1 IAV [2] it was believed to have a high pandemic potential.

We developed a bioinformatics platform for the assessment of the pandemic potential of IAV based on the informational spectrum method (ISM). The ISM is a virtual spectroscopy method for investigating protein-protein interactions and for analyzing structure/function of proteins (for review see [3] and references therein). The ISM platform was used to analyze H5N1, H1N1 and H3N2 subtypes and to assess their pandemic potential [4–6]. Here, we used the ISM platform to analyze A/swine/Guangxi/18/2011 and A/swine/Guangdong/104/2013 SIV. Our results revealed a strong human propensity and a very high pandemic potential of SIVs represented by the A/swine/Guangdong/104/2013 virus. We predict mutations which would further enhance human propensity of these viruses and propose conserved antigenic determinants which could serve as a component of the prepandemic vaccine. Finally, we propose a bioinformatics tool, which can be used to further monitor the evolution of SIV towards a pandemic virus.

## Material and Methods

### Virus

All hemagglutinin subunit 1 (HA1) sequences of H1N1 IAV in the GISAID database were included [7].

### Informational spectrum method (ISM)

As previously described in detail [3], the amino acid sequence of a protein is represented as a linear array of N terms, each amino acid or term with its own weight. The weight assigned to a residue corresponds to its electron-ion interaction potential (EIIP) (Table 1). The resulting numerical sequence is subjected to a discrete Fourier transformation, which is defined as:

$$X(n)=\sum_{m=1}^{N}x(m)e^{-i2\pi n(m-1)/N},\;n=1,2,\ldots ,N/2$$ (1)

where x(m) is the m-th member of a given numerical series, N is the total number of points in this series, and X(n) are discrete Fourier transformation coefficients. These coefficients describe the amplitude, phase and frequency of sinusoids comprising the original signal. The absolute value of complex discrete Fourier transformation defines the amplitude spectrum and the phase spectrum. The complete information about the original sequence is contained in both spectral functions. However, in the case of protein analysis, the relevant information is presented in an energy density spectrum, which is defined as follows:

$$S(n)=X(n)X^{*}(n)=|X(n){|}^{2},\;n=1,2,\ldots ,N/2$$ (2)

**Table 1:** The electron- ion interaction potential (EIIP) of amino acids.

Amino acid	EIIP [Ry]
Leu	0.0000
Ile	0.0000
Asn	0.0036
Gly	0.0050
Glu	0.0057
Val	0.0058
Pro	0.0198
His	0.0242
Lys	0.0371
Ala	0.0373
Tyr	0.0516
Trp	0.0548
Gln	0.0761
Met	0.0823
Ser	0.0829
Cys	0.0829
Thr	0.0941
Phe	0.0946
Arg	0.0959
Asp	0.1263

This describes sequences as discrete signals. It is assumed that their points are equidistant with the distance d = 1. The maximal frequency in a spectrum defined in this way is F = 1/2d = 0.5. The frequency range is independent of the total number of points in the sequence. The total number of points in a sequence influences only the resolution of the spectrum. The resolution of the N-point sequence is 1/n. The n-th point in the spectral function corresponds to a frequency f(n) = nf = n/N. Thus, the initial information defined by the sequence of amino acids can now be presented in the form of the informational spectrum (IS), representing the series of frequencies and their amplitudes.

The IS frequencies correspond to the distribution of structural motifs with defined physicochemical properties determining a biological function of a protein. When comparing proteins, which share the same biological or biochemical function, the ISM technique allows to detect of code/frequency pairs which are specific for their common biological properties, or which correlate with their specific interaction. This common informational characteristic of sequences is determined by cross-spectrum or consensus informational spectrum (CIS). A CIS of M spectra is obtained by the following equation:

$$C(j)=\prod_{i=1}^{M}S(i,j),\;j=1,2,\ldots ,N/2$$ (3)

where S(i,j) is the j-th element of the i-th power spectrum and C(j) is the j-th element of CIS. Thus, CIS is the Fourier transform of the correlation function for the spectrum. In this way, any spectral component (frequency) not present in all compared informational spectra is eliminated. Peak frequencies in CIS are common frequency components for the analyzed sequences. A measure of similarity for each peak is a signal-to-noise ratio (S/N), which represents a ratio between signal intensity at one particular IS frequency and the main value of the whole spectrum. If the CIS for a group of proteins, with different sequences results in strictly defined peak frequencies, it means that the primary structures of these proteins encode the same information. It has been demonstrated that: (1) such a peak exists only for the group of proteins with similar biological functions; (2) no significant peak exists for biologically unrelated proteins; (3) peak frequencies are different for different biological functions. Furthermore, it was shown that the proteins and their targets (ligand/receptor, antibody/antigen, etc.) have the same characteristic frequency in common. Thus, it can be postulated that IS frequencies characterize not only the general function but also the recognition and interaction between a particular protein and its target. Once the characteristic frequency for a particular protein function/interaction is identified, it is possible to utilize the ISM approach to predict the amino acids in the sequences, which predominantly contribute to this frequency and which are likely to be crucial for the observed function as well as to design peptides having desired biological function.

### ISM-based phylogenetic analysis

We used ISM-based phylogenetic algorithm ISTREE [8,9] to analyze hemagglutinin subunit 1 (HA1) from H1N1 SIV (for access to ISTREE, we refer the reader to http://istree.bioprotection.org). Using ISM distance measure *d*_*2*_ defined as the ratio of specific frequencies correlating with human and swine propensity of H1N1 AIV [5], we generate phylogenetic tree by the following algorithm:

1. For each sequence calculate its spectrum:
  1.1 Convert amino acid sequence into signal with EIIP values.
  1.2 Decrease signal to zero mean.
  1.3 Zero-padding to length of the longest signal, to set the same resolution to all spectra.
  1.4 Apply Fast Fourier Transformation to signal to generate energy density spectrum.
2. Calculate the distance matrix with the distance measure between sequences *X*_*1*_ and *X*_*2*_ defined as:
  $$d_{1}(X_{1},X_{2})=\left|\frac{A_{1}(F_{1})}{A_{1}(F_{2})}-\frac{A_{2}(F_{1})}{A_{2}(F_{2})}\right|$$ (4)
  where *A*_*1*_(*F*_*1*_) and *A*_*2*_(*F*_*1*_) are amplitudes on frequency *F*_*1*_ = 0.295; *A*_*1*_(*F*_*2*_) and *A*_*2*_(*F*_*2*_) are amplitudes on frequency *F*_*2*_ = 0.055 in informational spectra on sequences *X*_*1*_ and *X*_*2*_ respectively.
3. Construct the Unweighted Pair Group Method with Arithmetic Mean (UPGMA) tree.

The Maximum Likelihood (ML) tree, based on Multiple Sequence Alignment (MSA), was constructed with MEGA5 software package [10], using Jones-Taylor-Thornton (JTT) model and Nearest-Neighbor-Interchange (NNI) heuristic method.

### Calculation of the sequence alignment score

The scoring scheme consists of character substitution scores (i.e. score for each possible character replacement) plus penalties for gaps. The alignment score is the sum of substitution scores and gap penalties and it reflects goodness of alignment. For calculation of the alignment core the portal http://www.genome.jp/tools/clustalw/ was used.

## Results

According to the ISM concept recognition and targeting between biological molecules interacting at distances >5Å, is characterized by the common frequency component in their Informational Spectrum (IS). The strength of this interaction is represented by the amplitude value at this frequency [3]. Both, human-type and swine-type receptors bind IAV by α2,6 linked sialic acid, (α2,6-SA). Which of these two receptor types will preferentially bind the virus is determined by the properties of swine and human proteins carrying α2,6-SA. As shown before HA1 of SIV strains that predominantly interact with the swine receptor are characterized by the IS frequency F(0.055) [5]. In contrast HA1 from SIV strains that preferentially infect humans are characterized by the frequency F(0.295) [5]. Thus, the amplitude values of F(0.055) and F(0.295) can be used as indicators of virus propensity (swine versus human). For instance, an increase in amplitude at F(0.295) and a concomitant amplitude reduction at F(0.055), indicates an enhanced propensity of SIV to interact with the human receptor [5]. Thus a ratio of amplitude values A(0.295)/A(0.055) > 1 suggests that SIV has a higher human propensity. Conversely, A(0.295)/A(0.055) < 1 suggests that SIV preferentially binds swine receptor. Although the higher value A(0.295)/A(0.055) suggests increased pandemic potential of SIV, this is necessary but not sufficient condition for pandemic virus. Other viral properties also play important roles in determination of pandemic potential of influenza virus. It has also been shown that the efficacy of the influenza vaccine depends on the spectral similarity between antigens of the vaccine virus and circulating viruses [6].

**Fig 1:**
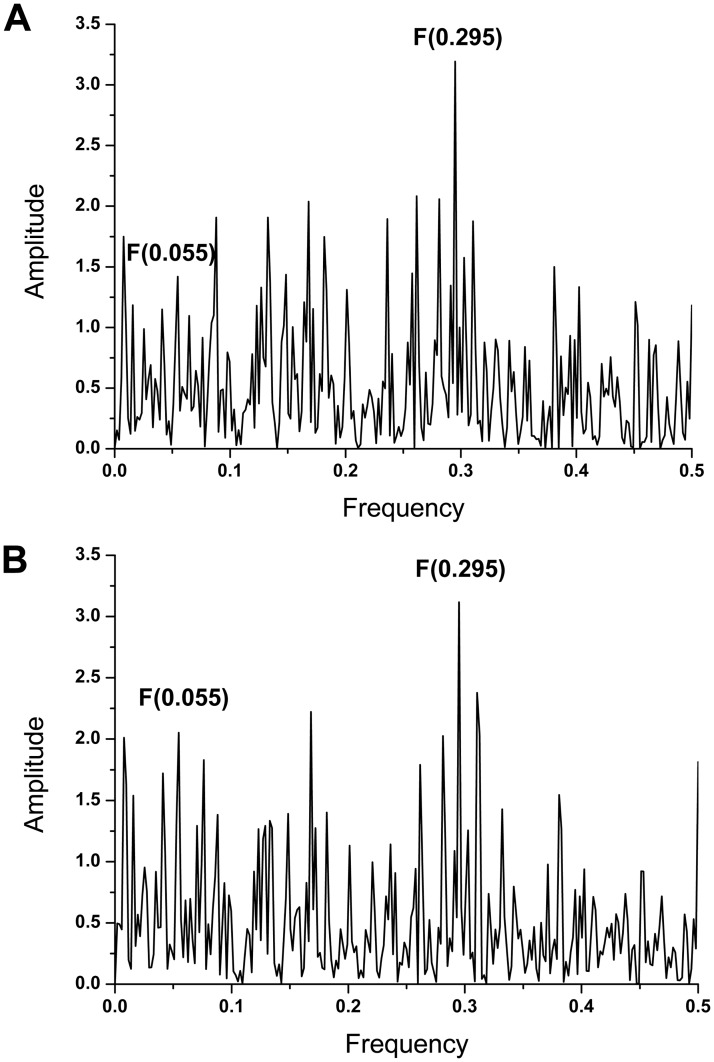
The informational spectrum of HA1 from SIV. (**A**) A/swine/Guangdong/104/2013 and (**B**) A/swine/Guangxi/18/2011.

Fig 1 shows the IS of HA1 from SIV A/swine/Guangxi/18/2011 and A/swine/Guangdong/104/2013. The amplitude ratios A(0.295)/A(0.055) calculated for these viruses are shown in Table 2. The amplitude ratio A(0.295)/A(0.055) was found to increase from 1.52 to 2.25 for A/swine/Guangdong/104/2013 in comparison to A/swine/Guangxi/18/2011. This very high amplitude ratio of A/swine/Guangdong/104/2013 suggests an increased possibility to interact with the human receptor.

**Table 2:** The amplitude values on IS frequencies F(0.055) and F(0.295) calculated for HA1 of SIV and the pdmH1N1 vaccine virus A/California/07/2009.

Virus	A(0.055)	A(0.299)	A(0.299)/A(0.055)
A/swine/Guangdong/104/2013	1.418	3.193	2.25
A/swine/Guangxi/18/2011	2.052	3.117	1.52
A/California/07/2009	2.397	2.644	1.10
A/Hebei-Yuhua/SWL1250/2012	1.948	3.043	1.56
A/swine/Guangdong/104/2013 with mutation D77N	1.144	3.556	3.11

**Fig 2:**
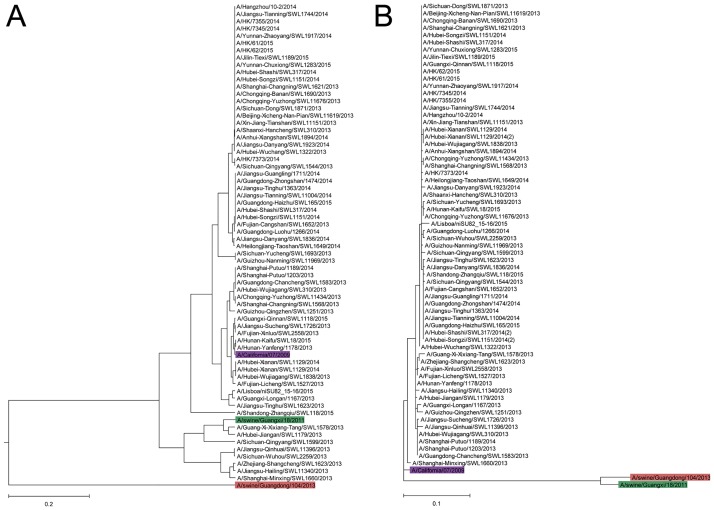
Phylogenetic comparison of pdmH1N1 IAV detected between 2011 and 2015 in China, and SIV A/swine/Guangxi/18/2011 and A/swine/Guangdong/104/2013. (**A**) The ISM-based phylogenetic tree; (**B**) the sequence similarity-based ML phylogenetic tree. The phylogenetic trees are created using HA1 protein sequences.

In an earlier study it was found that 3.6% of children and 13.4% of elderly adults had neutralization antibodies against the A/swine/Guangxi/18/2011 virus, but none of them had such antibodies against the A/swine/Guangdong/104/2013 virus [2]. As can be seen in the ISM-based phylogenetic tree (Fig 2A), the HA1 from the A/swine/Guangxi/18/2011 virus belongs to the group of seasonal pdmH1N1 isolated in 2011–2015 in China, while the A/swine/Guangdong/104/2013 virus is separated from this group. Previously it was demonstrated that the common IS frequency component in the spectra of proteins indicates their immunological cross-reactivity (see [11] and references therein). From this point of view, our result suggests potential antigenic cross-reactivity between seasonal pH1N1 viruses and A/swine/Guangxi/18/2011, but not with the A/swine/Guangdong/104/2013 virus. Of note is that the sequence similarity-based phylogenetic analysis cannot discriminate A/swine/Guangxi/18/2011 and A/swine/Guangdong/104/2013 viruses (Fig 2B).

**Fig 3:**
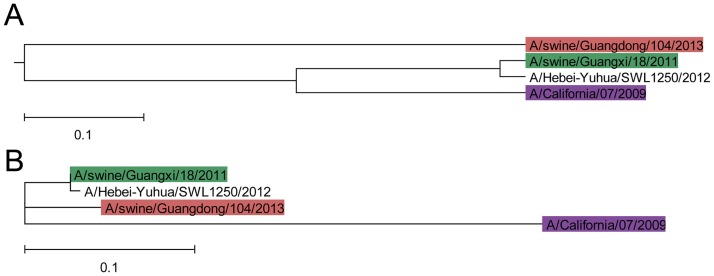
Phylogenetic comparison of A/swine/Guangxi/18/2011, A/swine/Guangdong/104/2013 and A/Hebei-Yuhua/SWL1250/2012 and pdmH1N1 A/California/07/2009 virus. (**A**) The ISM-based phylogenetic tree; (**B**) The sequence similarity-based ML phylogenetic tree.

Despite the low vaccination coverage in China against seasonal IAV including pdmH1N1 below 2% [2], an implication of immunity due to seasonal vaccination in cross-reactivity of humans against the A/swine/Guangxi/18/2011 virus, cannot be excluded [2]. Cross reactivity was reported between SIV A/Hebei-Yuhua/SWL1250/2012 and the highly homologous HA1 from A/swine/Guangxi/18/2011, as well as with the vaccine virus A/California/07/2009 [12]. The ISM-based phylogenetic tree of HA1 (Fig 3A) shows indeed that A/Hebei-Yuhua/SWL1250/2012, A/swine/Guangxi/18/2011 and A/California/07/2009 group together, while the A/swine/Guangdong/104/2013 is separated. In contrast, the sequence similarity-based tree (Fig 3B) shows that all EA H1N1 SIVs group together and are separated from the vaccine virus. These results of Fig 3 are indicative of similar interacting properties of HA1 of A/swine/Guangxi/18/2011 and A/California/07/2009 viruses, despite significant sequence differences (alignment score of 72.5). On the other hand, HA1 from A/swine/Guangxi/18/2011 and A/swine/Guangdong/104/2013, despite sequence similarity (alignment score 93), show different interacting profiles represented by their distinct IS characteristics.

The computer scanning survey of the HA1 amino acid sequence of the A/swine/Guangdong/104/2013 virus showed that the main contribution to the information represented by the frequency F(0.295) comes from a domain comprising residues 232–267 (denoted VIN2,1) of the mature protein (H1 numbering). This domain VIN2,1 is located closer to the receptor binding domain (RBD) than the corresponding domain VIN1 of pdmH1N1 viruses [5]. This suggests that the swine virus A/swine/Guangdong/104/2013 may be more efficient in recognition and targeting of human receptor than seasonal pdmH1N1 viruses. The domain VIN2,1 which is highly conserved, because of its important role in virus/receptor interaction, represents a candidate target for differential diagnostics, therapies and vaccines.

## Discussion

The long-range interaction, which affects the number of successful collisions between virus and receptor, is only the first step of their interaction. The second step, the non-covalent binding of HA1 RBD to the receptor, is determined by their structural (3D) complementarity. Absence of an efficient human-to-human transmission of A/swine/Guangdong/104/2013 suggests that the RBD of this virus may not yet be fully adapted to the human receptor. It cannot be excluded that this virus will evolve to bind more efficiently to the human receptor. In this case A/swine/Guangdong/104/2013, which is already well adapted for interacting with the human receptor, has the potential to become a new pandemic virus. Therefore, close monitoring of the evolution of SIV strains represented by the A/swine/Guangdong/104/2013 virus is warranted in order to predict and prevent the development and spread of a new pandemic.

Previously, we suggested that frequency F(0.236) in IS of HA1 from avian H5N1 IAV reflects the binding of this protein to the human receptor [4]. More recently it was experimentally confirmed that mutations which increase the amplitude of this frequency predicted by ISM, also increased binding of H5N1 HA1 to the human receptor [13]. Alanine scanning mutagenesis of HA1 of the A/swine/Guangdong/104/2013 performed *in silico* revealed that position D74 represents a hot spot for mutations which would most significantly increase the value of the amplitude ratio A(0.295)/A(0.055). By analogy with results obtained for H5N1 viruses [5,8,13], it could be expected that these mutations will further increase the human propensity of the A/swine/Guangdong/104/2013 virus. SIV strains represented by A/swine/Guangxi/18/2011 already have N74, suggesting a high probability for this mutation to occur also in SIV represented by the A/swine/Guangdong/104/2013 as a consequence of viral adaptation. Introduction of mutation D74N into HA1 of these viruses increases the amplitude ratio A(0.295)/A(0.055) by 39% (Table 2) which could significantly increase their human propensity.

Monitoring of the evolution of SIV toward possible pandemic viruses represents an important measure in influenza pandemic preparedness. For this reason, we established the ISTREE service for monitoring of SIV evolution which is freely available on http://biomedprotection.com/iav_mon.php.

### Limitations

Determination of the statistical significance of the value/change of the A_F(human)_/A_F(animal)_ ratio in assessment of the influenza virus tropism, would be useful. As it is pointed, the ratio A_F(human)_/ A_F(animal)_ >1 is necessary but not sufficient condition for the human propensity of influenza viruses because other viral proteins also play important roles in the virus/host interaction. For this reason, influenza virus with A_F(human)_/A_F(animal)_ >1 will not be obviously the “human virus”, independently how this ratio is high. Contrary, influenza virus with A_F(human)_/A_F(animal)_ <1 can not be the “human virus” even if its other proteins are well adopted to the human host. For this reason, it is not possible to establish direct correlation between the A_F(human)_/A_F(animal)_ value and the human propensity of influenza viruses.

## Conclusions

The presented *in silico* analysis showed that HA of the A/swine/Guangdong/104/2013 virus acquired mutations which could increase its human tropism. This is potentially indicative of a significant pandemic potential of the A/swine/Guangdong/104/2013 virus, stressing the urgent need to contain this virus. Our results also suggest an antigenic determinant which could be included in a prepandemic vaccine against A/swine/Guangdong/104/2013 SIV. Finally, the freely available bioinformatics platform for monitoring of the evolution of SIV toward possible pandemic viruses is established (http://www.vin.bg.ac.rs/180/tools/iav_mon.php; http://www.biomedprotection.com/iav_mon.php).
